# Complications of Subcision for Acne Scarring: Experience From Clinical Practice and Review of the Literature

**DOI:** 10.1111/jocd.16629

**Published:** 2024-11-03

**Authors:** Cong Sun, Davin Lim

**Affiliations:** ^1^ Mater Hospital Brisbane Brisbane Queensland Australia; ^2^ Cutis Clinic Brisbane Queensland Australia

**Keywords:** acne scarring, complications, subcision

## Abstract

**Background:**

Subcision is a surgical technique frequently used in the management of atrophic acne scars. The advent of new instruments, which includes sharp, blunt and energy assisted, have increased the efficacy of the procedure. The aim of this article was to review the safety of subcision in view of the new development in technology.

**Objective:**

To review the safety of subcision procedure for acne scarring and to provide clinicians with both evidence‐based and practical information regarding the complications that can be associated with this procedure.

**Methods:**

A search through MEDLINE and Google Scholar was conducted for articles from January 2000 to January 2023 that involves subcision as a monotherapy for the treatment of acne scarring.

**Results:**

Ten articles involving subcision monotherapy were identified. The main complications of subcision were pain/tenderness, bruising, infection, formation of subcutaneous lumps, and dyspigmentation. Recommendations on how to minimize the complications from subcision have also been provided based on the clinical experience of the authors.

**Conclusion:**

Subcision is a safe treatment for acne scarring and clinicians need to be aware of the associated complication which occurs more with sharp instrumentation.

## 1 Introduction

Subcision, which is also known as subcutaneous incisionless surgery, is a modality of treatment used in a variety of skin conditions including atrophic acne scar, cellulite, and rhytides. This procedure was first described in 1995 by Orentreich, which offered a minimally invasive treatment for a variety of different conditions [1, 2, 3]. The two proposed mechanisms of subcision included detaching the fibrotic strands associated with scarring and new connective tissue formation through local trauma [4]. Since the advent of this procedure, many modifications have come forward to refine the technique. Traditionally, subcision is a safe procedure with few associated complications including bleeding, bruising, and temporary pigmentation. The purpose of this review was to explore the rates of complication associated with this procedure in view of the new development in this method of scar revision.

## 2 History and Evolution of Subcision

Orentreich introduced the concept of “subcutaneous incisionless” surgery in 1995 in a case series involving three patients. The study introduced a technique using a tribeveled 22‐gauge hypodermic needled inserted under the skin and maneuvered under the defect and make subcuticular cuts to remove the subcutaneous fibrotic strands and promote collagen synthesis through controlled local trauma [1].

Subsequently, subcision procedure has been defined as the insertion of an instrument into the skin to manually detach scar tissue within the dermis to restore the contour of the skin. Subcision can be done in “superficial” or “deep fashion.” Shallow scars may only require subcision into the depth whereas deeper scars such as rolling scars may need to have subcision deeper into the subdermal level due to fibrous anchoring to the subcutis [5].

Since then, several modifications have been made to refine the technique [2]. Alam, Omura, and Kaminer [6] showed subcision using Nokor needles, an 18‐gauged tribeveled hypodermic needle. This device allows triangular tip allows smooth and thorough separation of the subcutaneous fibrotic strands and attachments [7]. Additionally, cataract blade has also been promoted as a novel tool for subcision [8].

Although needle subcision is a practical method, the rate of recurrence is common and overall efficacy is moderate [9]. Nilforoushzadeh et al. [9] pioneered a new technique using a blunt 18‐gauge cannula in a randomized study which showed higher efficacy compared with the traditional needle subcision in the control group. A recent development involved a combination of a blunt cannula with a radiofrequency probe for easier destruction of fibrous strands with reduced bleeding [10].

Subcision performed with high‐gauge hypodermic needles or cannulas often perforate the fibrotic strands but does not fully sever them to release the resulting depression. Wire scalpel as an alternative tool for subcision was first introduced by Sulamanidze et al. [11] to allow back‐and‐forth sawing motion to release the fibrotic tethering. The original study by Sulamanidze involved wire subcision for age‐related wrinkles; currently, there have been no studies utilizing the same technique for acne scar revision.

The latest addition are blunt blade instruments, commonly known as a liberator. This instrument resembles a long screwdriver with a notched end to capture fibrotic tethering [12]. Clinicians can locate the subcutaneous tethering by the resistance to the motion of the instrument [12].

## 3 Material and Methods

A literature search was performed on all article up to January 2023. The search was conducted through MEDLINE (OVID) with the keywords “subcision” and “acne” as a MESH term. This review was limited to procedures that were limited to subcision as a monotherapy to focus on the complications that solely originate from subcision alone rather than potential complications from combination therapy. Split‐face studies involving subcision and other modalities of scar revision on the contralateral side of facial skin were included provided only one treatment modality is used on each side of the skin. Editorials, conferences papers, commentaries, and letter to editors were also excluded from this study.

## 4 Results

At the conclusion of the literature search, 10 studies involving subcision monotherapy were identified. Out of the 10 studies, 5 were split face studies with subcision and other modalities of scar revision performed on separate side of the facial skin with crossing over. Five studies were small case series/prospective cohort studies. Two studies were nonblinded randomized control trials comparing two different subcision tools.

## 5 Discussion

**FIGURE 1:**
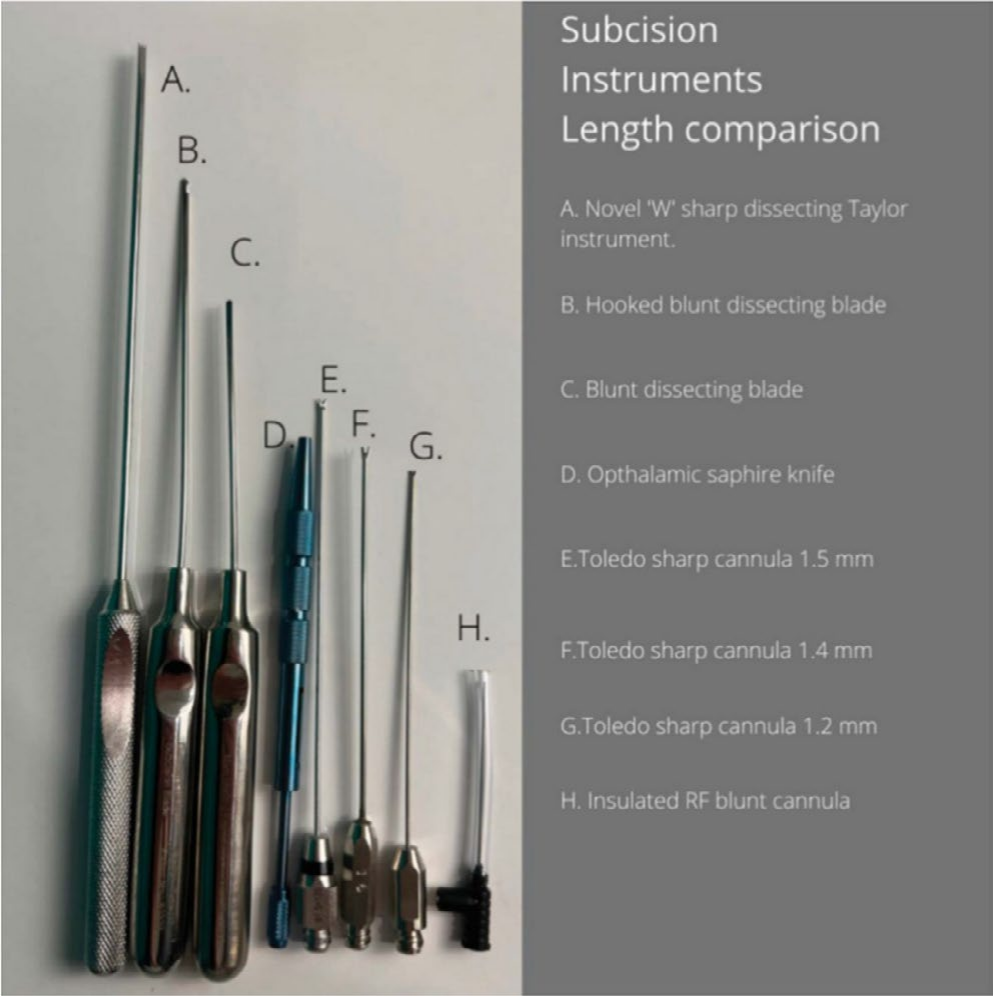
Common instruments used in subcision for acne scarring.

Within the current literature; pain/tenderness, bruising, infection, formation of subcutaneous lumps, and dyspigmentation are commonly reported side effects of subcision therapy for acne scar revision. Most of the complications often resolve spontaneously postprocedure without the requirement of further interventions. Although the original paper by Orentreich reported hypertrophic scarring as a complication of subcision, the incidence of such is seldom seen within the literature (Figure 1).

The current literature predominantly consists of small case series or prospective cohort studies. The reporting of complications associated with subcision in these studies is inconsistent and unclear. Quantitative data regarding the rate of complication is not infrequently missing from the studies and the reporting of complications is largely descriptive. The authors, therefore, will conduct the discussion section of this paper using a combination of existing literature and personal experience from conducting subcision. The evidence is summarized in Table 1.

**TABLE 1:** Summary of complications associated with subcision in the literature.

Author's first name	Year of publication	Number of participants	Instrument	Complications
Ramadan	2011	20	1.5‐in. NoKor Admix needle	Erythema (5), hyperpigmentation (3), and hypopigmentation (3)
Alam	2006	40	18‐gauge Nokor needle	Qualitative description of ecchymoses, edema, and erythema
Nilforoushzadeh	2020	100	18‐gauge cannula versus 27‐gauge needle	Infection (0 vs. 4, *p* > 0.05), Scar formation (0 vs. 18, *p* > 0.05)
Nilforoushzadeh	2022	9	Endo‐RF subcision	Qualitative description of mild erythema and oedema
Barikbin	2017	18	Blunt subcision blade	Postsubcision pain (7), mild‐to‐moderate swelling (5), periorbital ecchymoses (3)
Nilforoushzadeh	2015	8	18‐gauge spinal needle Cannula	Qualitative description of edema and inflammation
Asilian	2018	28	Nokor needle versus blunt blade subcision	Bruising (14 vs. 7, *p* = 0.053), Hematoma (3 vs. 0, *p* = 0.236), Subcutaneous nodule (4 vs. 0, *p* = 0.038), Scarring (1 vs. 0, *p* = 0.313) Complications also included hyperpigmentation, hypopigmentation, bleeding, infection, and secretion but did not occur in either the Nokor needle or blunt blade subcision groups
Gheisari	2019	34	Nokor Needle versus Blunt blade subcision	Qualitative description of ecchymosis and nodule formation
Ebrahim	2021	46	Tribevel hypodermic needle versus cannula subcision	Statistically significant difference between Needle and Cannula group in terms of pain (*p* < 0.001), edema (*p* < 0.001), and ecchymosis (*p* < 0.001)
Kaur	2019	21	Radiofrequency‐assisted subcision versus conventional cannula subcision	Qualitative description of swelling, bruising, and infection

### 5.1 Infection

Infection is an uncommon event following subcision and has been described after subcision surgery. A randomized control trial of cannula versus 27G hypodermic subcision (50 patients per group) revealed four cases of infection in the needle group, compared to 0 in the subcision group [13].

**Recommendations from Author:**

1. Avoid subcision in active areas of infection.
2. Adhere to aseptic technique, consider antiviral prophylaxis for patients with a history of HSV.

Comment: The role of prophylactic antibiotics is unclear. Wide dissection of the dermal subcutaneous junction within a cosmetic unit increases tissue dead space, with a potential for infection. For extensive subcision, the author prescribes doxycycline 50 mg for 7–14 days as both bacterial prophylaxis and an anti‐inflammatory; however, the efficacy of this requires further investigation.

### 5.2 Intra and Postoperative Bleeding

**FIGURE 2:**
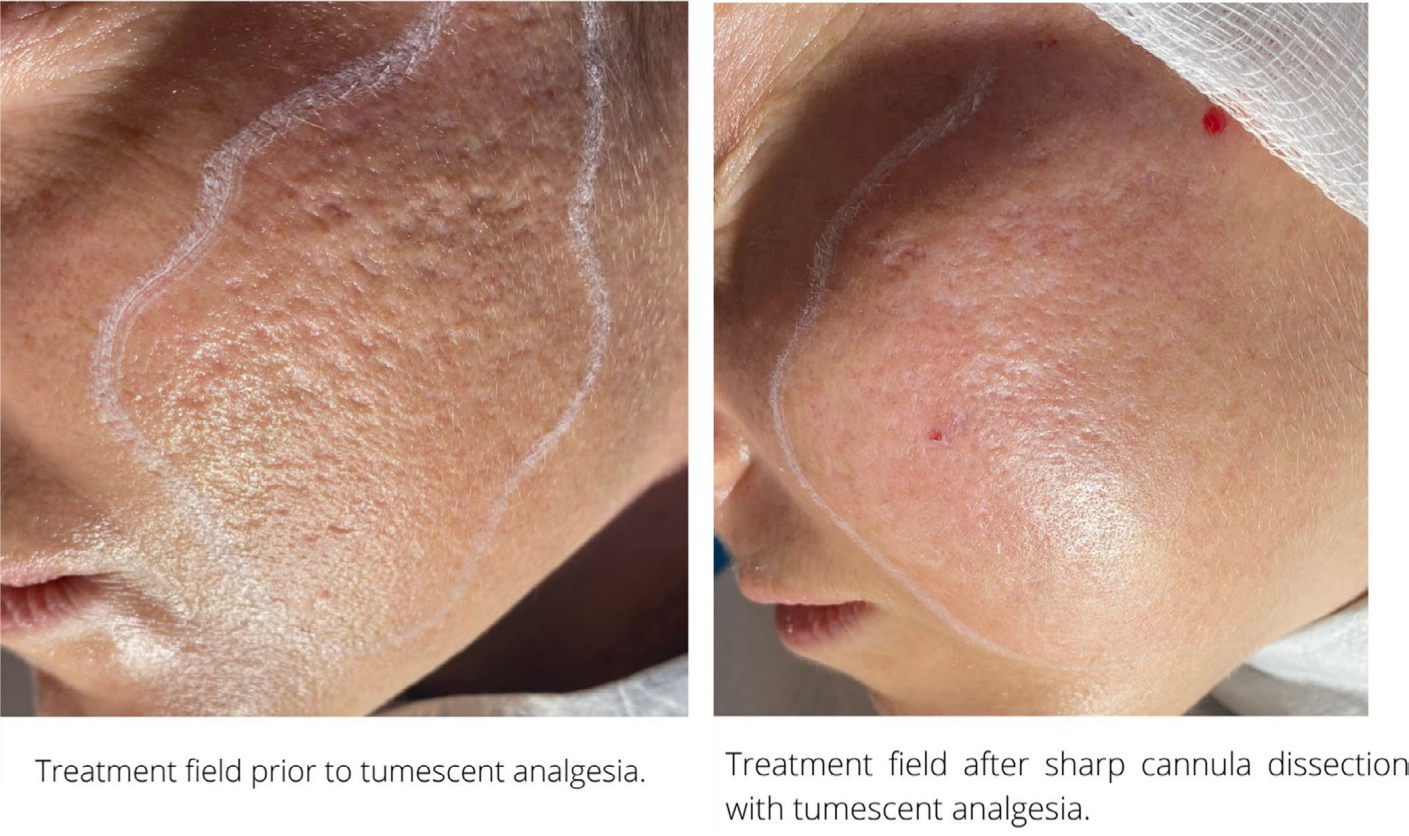
Tumescent infiltration of treatment field.

**FIGURE 3:**
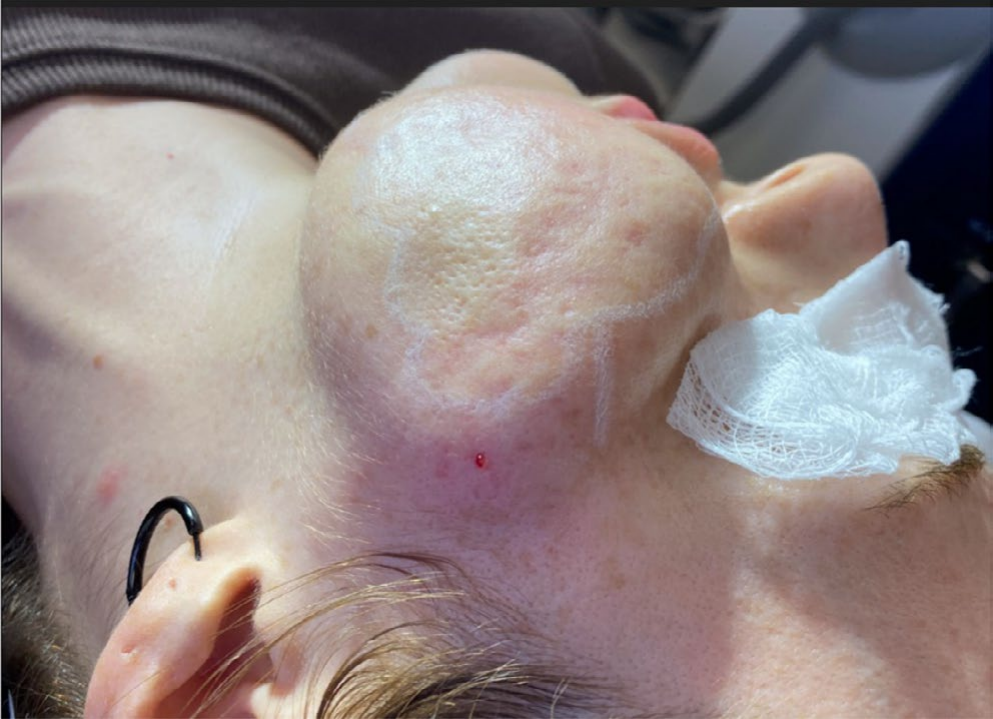
Author typically injects 50 mL of tumescent anesthetic per side. This aids hemostasis, reduces lidocaine toxicity, optimizes hydrodissection and protects the underlying structure.

**FIGURE 4:**
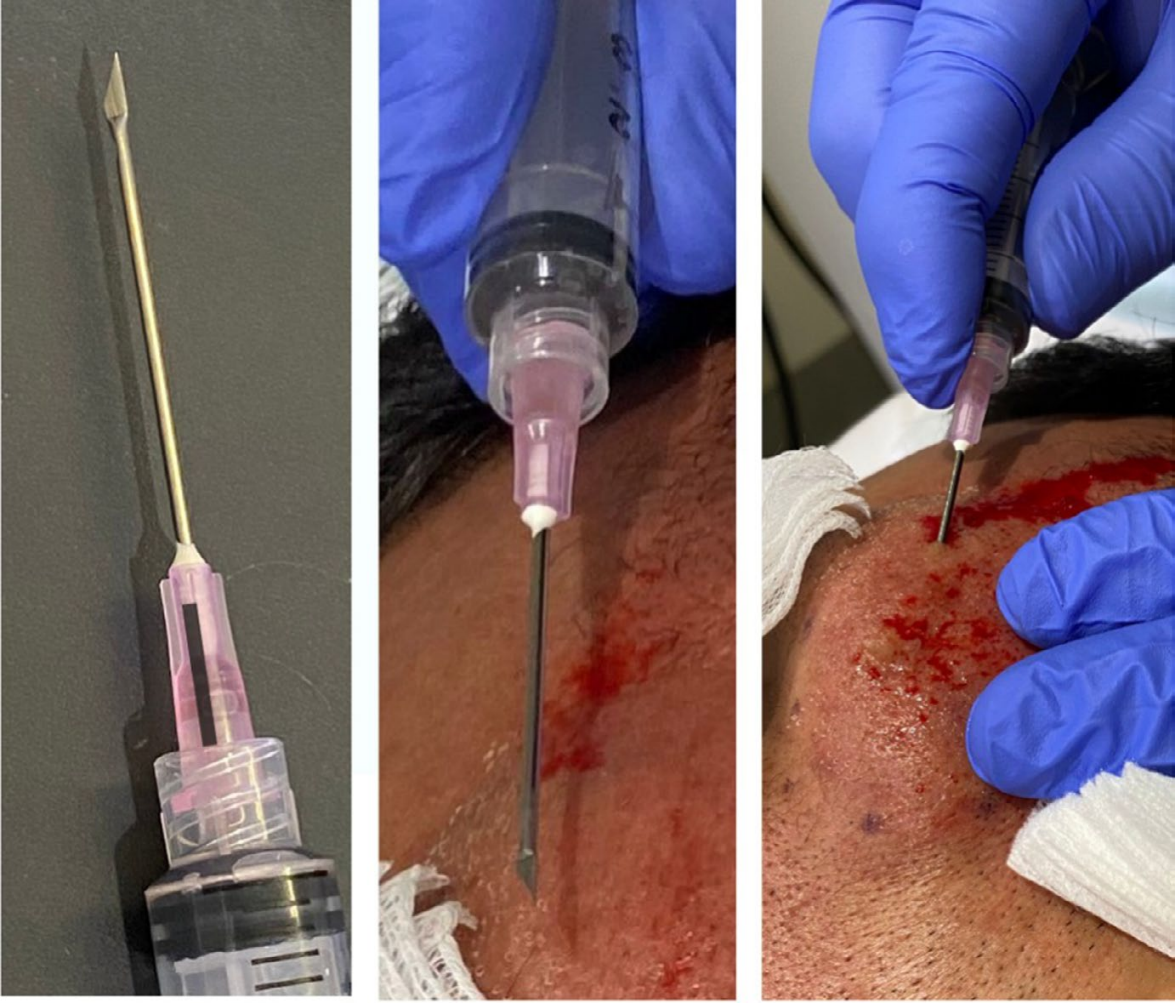
Orientation optimizes efficiency and reduce side‐effects. Luer lock syringe prevents the rotation of the cutting needles. Fingertip orientated to the cutting side.

Intra‐ and postoperative bleeding can be mitigated by the infiltration of tumescent local anesthetic with epinephrine. Volume ranging from 20 to 60 mL can be infiltrated per sides of the cheeks (Figures 2, 3, 4).

The risk of bleeding can also be mitigated by modifying the sharp needles. Khunger [14] reported a novel technique involving the modification of a sharp‐beveled needle with a 90° bend which allows the needle to cut parallel to the skin's surface. Modifying the orientation of the cutting edge of Nokor needle with a needle holder has also been reported as a measure to reduce subcision complications [7].

There is limited evidence that cannula subcision has lower risk of intraoperative and postoperative bleeding compared to needle subcision. In a small split‐face trial with 46 patients, Ebrahim showed statistical (*p* < 0.001) reduction in ecchymosis in the cannula group compared with the needle subcision group [15].

The choice of instruments also plays a role in intra and postoperative bleeding. Blunt instruments possess the advantage of potential reduction in the risk of neurovascular injury and minimizing the risk of posttreatment purpura. In a split‐face comparison study between Nokor needle and blunt blade subcision, Asilian et al. [16] found that in a cohort of 28 patients, the rate of bruising (17 vs. 8), hematoma (3 vs. 0), formation of subcutaneous nodules (4 vs. 0), and hyperpigmentation (2 vs. 0) were higher in the side of the face treated with Nokor needle compared to blunt blade. Statistical significance was only demonstrated in the difference in bruising between the two surgical instruments (*p* < 0.001). In 2022, Nilforoushzadeh et al. pioneered a new technique attaching a radiofrequency probe to the metallic end of subcision cannula as a promising method to reduce hematoma formation and postoperative bleeding. In a limit study of nine patients, the endo‐RF subcision method is associated with mild edema and erythema without the complication of purpura, hematoma, and subcutaneous nodules [10]. A larger randomized trial comparing radiofrequency‐assisted and conventional subcision was done in 2019 by Kaur et al. [17] with 21 participants but the complications including oedema, ecchymosis, and infection were only reported qualitatively with no comparison between treatment arms.

**Recommendation from Authors:**

1. Local or tumescent infiltration of local anesthetic and epinephrine for vasoconstrictive effect.
2. Careful choice of blunt versus sharp instrumentation.
3. Consider modifying sharp needles to minimize overpenetration and accurate dissection of appropriate plane.

### 5.3 Hematoma

**FIGURE 5:**
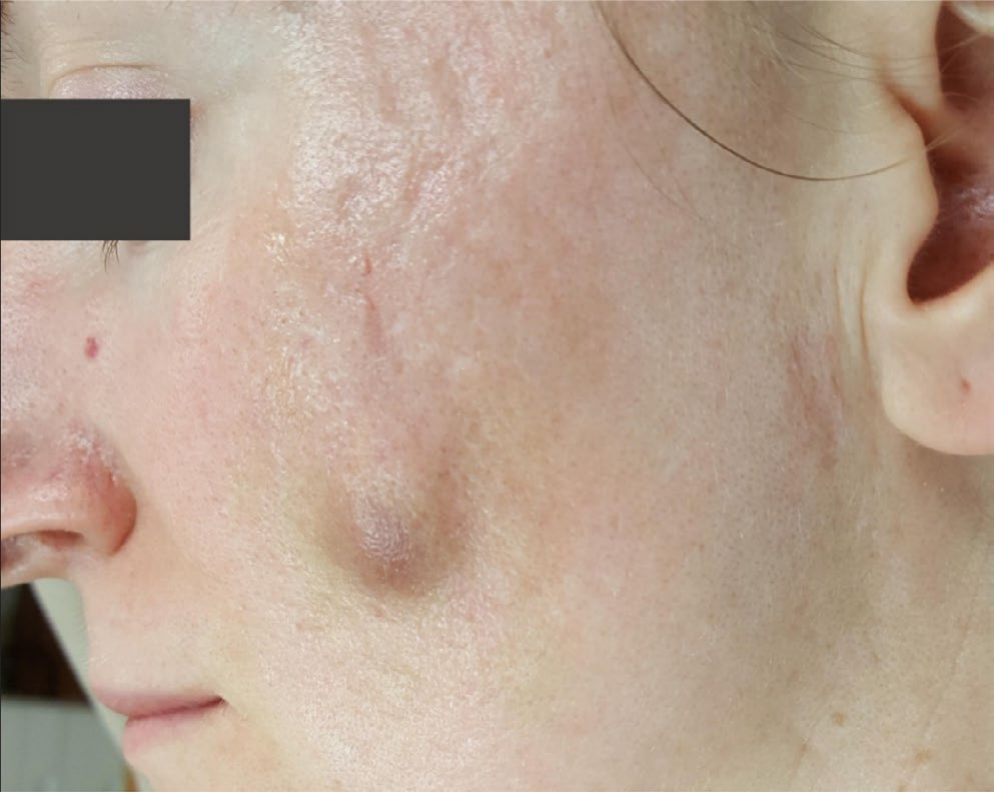
Hematoma secondary to Nokor subcision.

**FIGURE 6:**
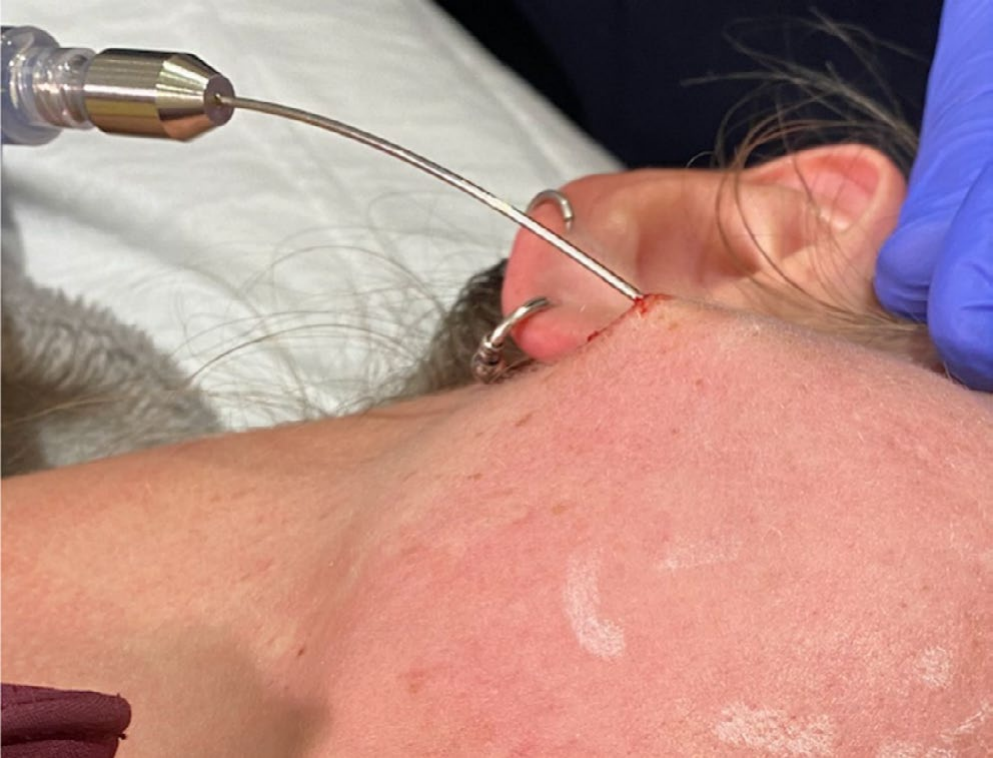
Long and flexible cannula minimizes the number of entry points requires and in turn reducing the chance of hyperpigmentation and iatrogenic scarring.

In most cases, hematomas postsubcision are idiosyncratic. Physicians can take steps to reduce the incidence of complications which can be related to patient factors, the procedure itself, and postoperative care. Subcision, with the use of a sharp dissecting instrument, is essentially blind undermining of a cosmetic unit. Surgeons must be vigilant regarding methods to reduce the rate of hematoma by taking in to consideration the aggression and extent of the procedure and the risk of intra and postoperative bleeding (Figures 5, 6). Patient factors such as medications which increase bleeding time or tendency for hemorrhage as well as agents which may alter blood pressure. Expanding hematomas need to be evacuated and drained which can be achieved with a roll sterile gauze inserted into the instrument entry site. Small hemorrhagic nodules may resolve over 1–2 months and larger clots can be treated with diluted intralesional corticosteroid, or 5‐fluorouracil.

In terms of the evidence within the current literature, the first paper involving blunt blade excision by Barikbin et al. [12] reported five cases (27.8%) of mild to moderate swelling, seven cases (38.9%) of postoperative pain/tenderness, and three cases (16.7) of ecchymosis. In a split‐face comparison study between Nokor needle and blunt blade subcision, Asilian et al. [16] found that in a cohort of 28 patients, the rate of bruising (17 vs. 8), hematoma (3 vs. 0), formation of subcutaneous nodules (4 vs. 0), and hyperpigmentation (2 vs. 0) were higher in the side of the face treated with Nokor needle compared with blunt blade. Statistical significance was only demonstrated in the difference in bruising between the two surgical instruments (*p* < 0.001).

**Recommendations from Authors:**

1. Evaluate procedural factor and patient factors prior to procedure.
2. Careful consideration of patient's risk of bleeding from medical comorbidities, medication, and blood pressure control perspective.
3. Evacuate expanding hematoma (e.g., rolls sterile pieces of gauze starting 1–2 cm from beyond the margin of the subcision area, toward the instrumentation entry site.)

### 5.4 Paresthesia and Neuralgia

The extensive network of sensory nerves of the face and neck and their innervation at the same level of dissection entails that paresthesia should be discussed with the patient prior to surgery.

Paresthesia is due to the transection of the sensory cutaneous branches of the trigeminal nerve. It is more commonly encountered in the forehead area as the branches of the supraorbital and supratrochlear nerves are superficially located. Extensive subcision of the cheeks may result in variable paresthesia as the superficial branches of the infraorbital and zygomaticofacial branches are transected [18].

**Recommendations from Authors:**

1. Care should be taken in the proximity of major neurovascular bundles.
2. A higher incidence of paresthesia can be expected with wide tip/larger diameter instrumentation. Consider the use of blunt instruments in the forehead areas.
3. In the majority of cases, paresthesia is self‐limiting with spontaneous improvement seen within 6–18 months. Permanence has been described.
4. Neuralgia is exceedingly rare following subcision.

### 5.5 Hyperpigmentation

**FIGURE 7:**
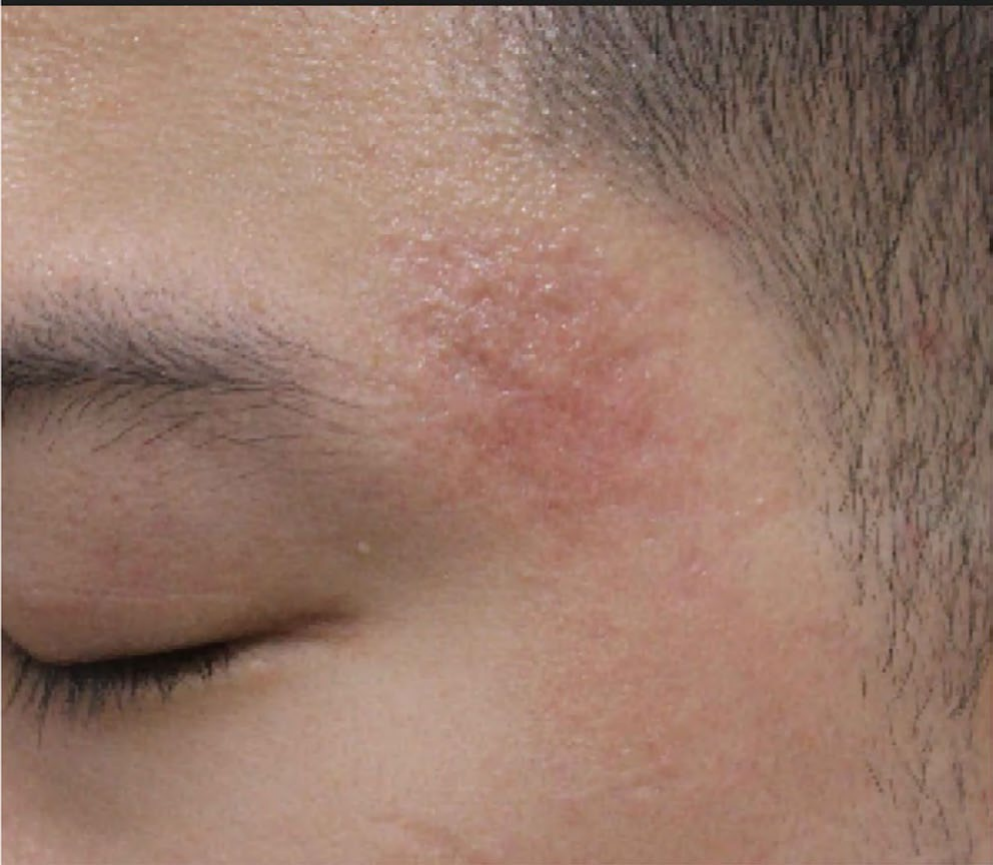
Fibroplasia and postinflammatory hyperpigmentation secondary to cannula subcision of the temple.

Postinflammatory hyperpigmentation (PIH) results from injury to the dermoepidermal junction (Figure 7). PIH postsubcision is primarily due to two factors. Firstly, the instrument entry line may produce hyperpigmentation and hypertrophic scarring. Less commonly, postinflammatory hyperpigmentation may be due to hemosiderin deposition from intra or postoperative bleeding and purpura. Some of the measures that can be taken include careful planning to reduce entry points for instrumentation, lateral entry points for triangulation of the area, longer instrument over shorter blades, careful hemostasis to reduce postprocedure purpura and PIH, and strict photoprotection postprocedure. The treatment for postinflammatory hyperpigmentation includes tyrosinase inhibitor, high factor SPF sunscreen in the correct amount and frequency, and picosecond lasers or 1027 thulium/diode to speed up resolution.

With regard to the existing literature, most of the studies currently shows that erythema and pigmentation as common transient complications of subcision [19]. There is evidence from two small split‐face comparative studies that blunt instrument subcision is associated with lower risk of hyperpigmentation compared with sharp instruments. A comparative study between cannula‐based subcision and needle method was done by Nilforoushzadeh et al. [9] in a cohort of 100 patients. Nilforoushzadeh et al. reported complications including bruising, swelling, postinflammatory hyperpigmentation, and scar formation. The difference in rate of complication between cannula‐based method and common needle subcision was statistically significant (*p* < 0.05); however, the study did not provide breakdown in terms of the difference between specific complications. In a small split‐face study with 34 patients undergoing subcision with blunt cannula and Nokor needle, Gheisari, Iranmanesh, and Saghi [20] ecchymosis, nodule formation, and oedema postsubcision. However, there were no reported quantitative data regarding rate of complication to compare the safety profile of the two treatment modalities.

**Recommendations from Authors:**

1. Reduce entry points for instrumentation.
2. If triangulation of the area is required consider lateral entry points.
3. Consider longer instrumentation over shorter blades and “point” subcision.
4. Ensuring homeostasis postoperatively will reduce post procedure purpura and PIH.
5. Advise strict photoprotection postprocedure.

### 5.6 Iatrogenic Scars/Exaggerated Fibroplasia

**FIGURE 8:**
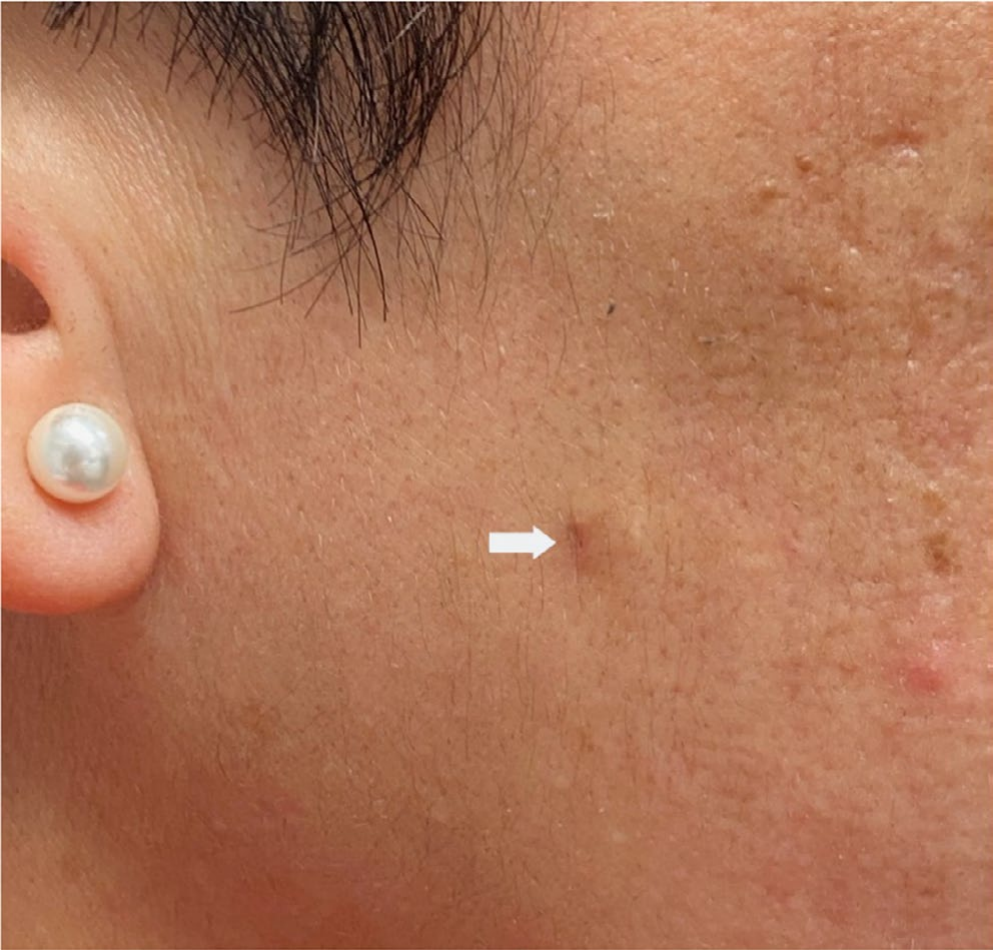
Hypertrophic and pigmented iatrogenic scar secondary to dovetail cannula.

**FIGURE 9:**
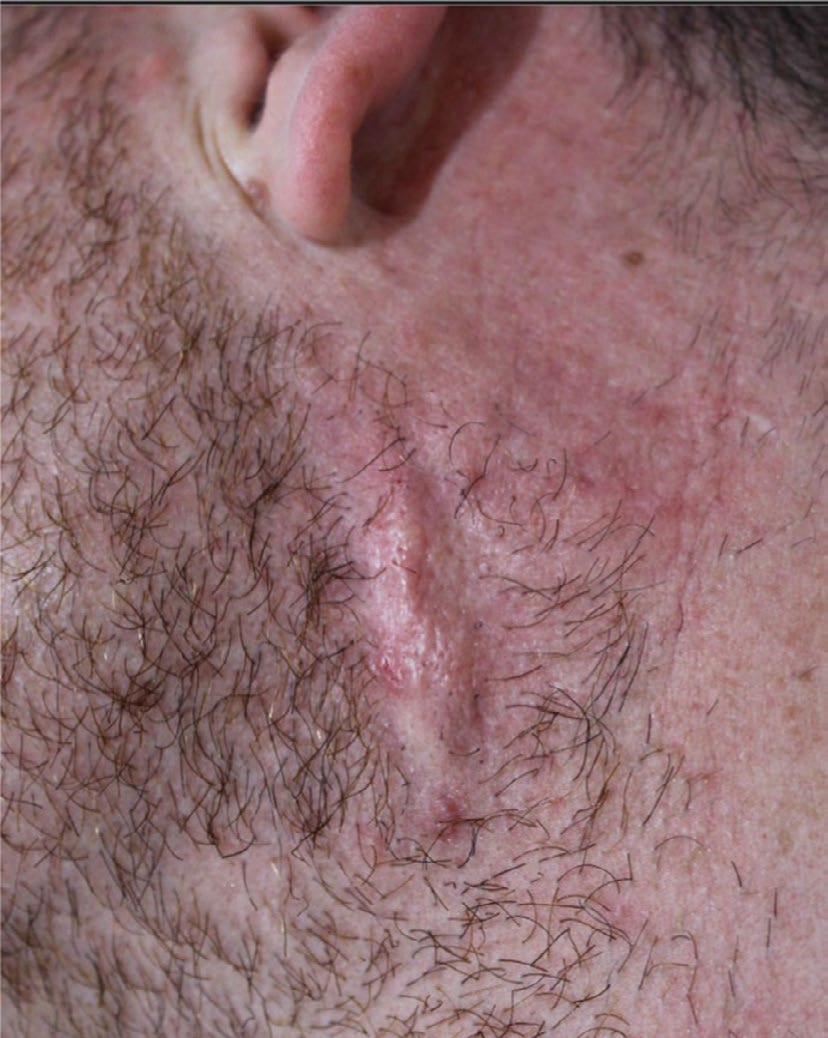
Hypertrophic scarring secondary to Nokor Subcision.

Iatrogenic scarring can occur at the instrumentation entry site (Figure 8, 9). Reactive fibroplasia, as defined by pathological increase in connective tissue, is relatively rare and need to be differentiated from hematomas. Studies have shown that patients treated with sharp needle excision are more prone to scar formation compared to blunt subcision [7].

**Recommendations from Authors:**

1. Careful planning of instrumentation entry sites can reduce the number of perforations. The use of longer cannula or novel instruments can maximize the area of subcision, as compared to “point” subcision from Nokor, cataract blades or hypodermic needles.
2. Consider a lateral entry point, classically 0.5–1.5 cm anterior to the tragus. This can reduce the visibility of scars compared to centrofacial entry points.
3. The use of cannulas over sharp subcision is associated with a lower incidence of fibroplasia. Less aggressive subcision may be indicated in those who exhibit a brisk fibroblastic response or with a personal/family history of hypertrophic or keloid scarring.
4. The author prescribes doxycycline 50 mg od for 4 weeks as an anti‐inflammatory agent. Further studies are required to ascertain efficacy.
5. Firm daily massage several minutes a day for several weeks can accelerate resolution of bumps.

### 5.7 Avulsion of Retaining Ligaments

**FIGURE 10:**
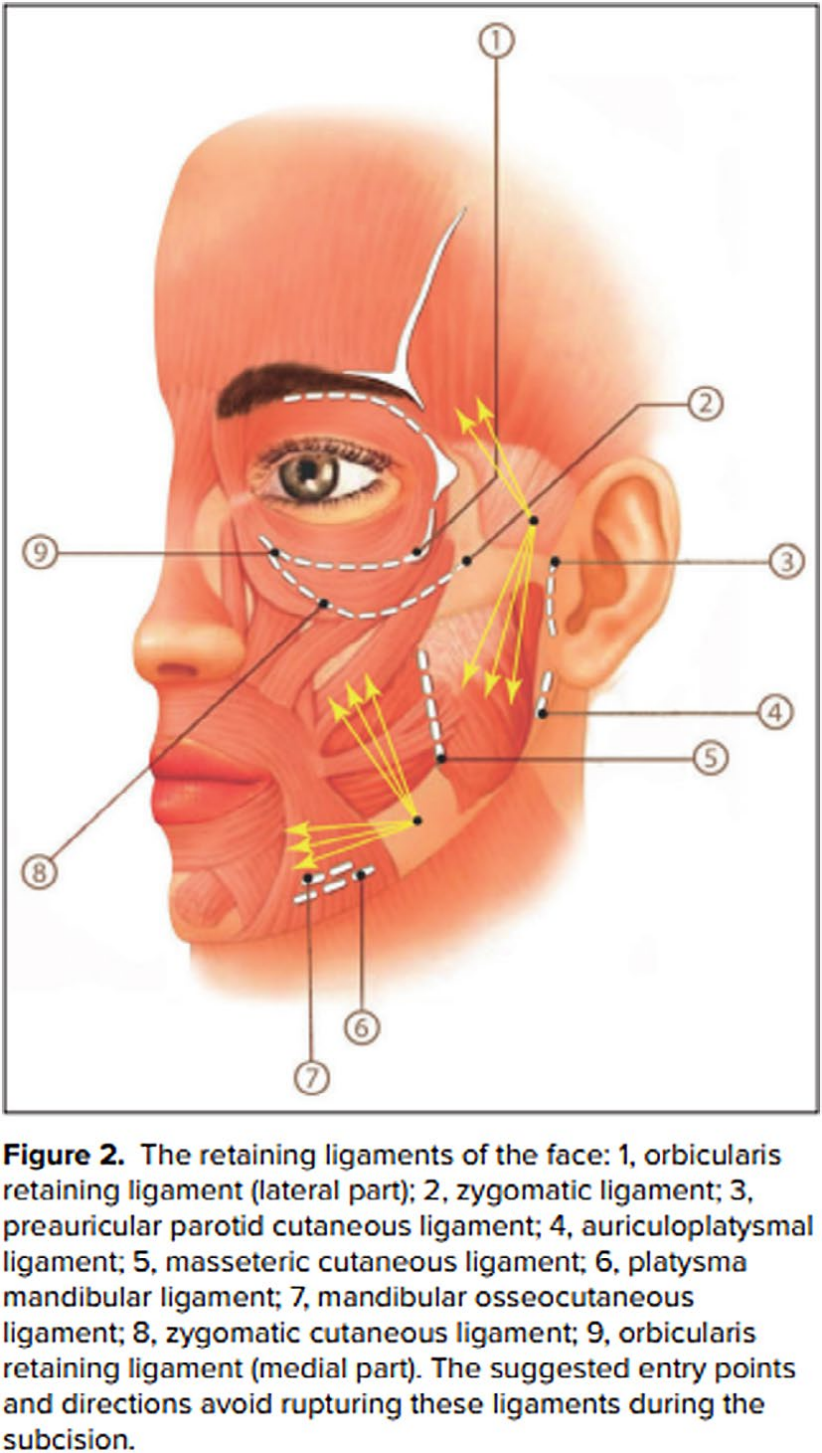
Retaining ligaments of the face. Courtesy of Araghi et al. [24].

The ligamentous of the face has a significant role in the mechanism of facial aging as they prevent laxity and sagging of the skin (Figure 10). The ligamentous apparatus of the face can be divided into true and false retaining ligaments [21]. The True retaining ligaments are structures that originate from the periosteum and attach to the fibrous septae and dermis. The true ligaments are located in the zygomatic, preauricular, mandibular, and orbital region of the face (The false ligaments attach the superficial musculoaponeurotic system to the deep fascia but has not connection to the periosteum and are thought to have a lesser role in facial aging and therefore sagging and skin laxity) [21]. The occur in constant anatomic locations and serve to separate facial compartments. Given their attachments in the hypodermis, the process of subcision often transects the fibrous strands in the subcutaneous layer, which may lead to facial sagging and laxity.

Careful planning and understanding of the origin and insertion of facial ligaments can minimize avulsion of these ligaments [24].

**Recommendations from Authors:**

1. Selection of multiple entry points to minimize collateral damage to retaining ligament of the face.
2. Consider the use of blunt instruments over sharp dissecting cannulas.
3. Limiting the extent of undermining during the procedure.
4. Consider multiple procedures to subcise extensive subdermal scarring.

### 5.8 Subcision and Isotretinoin

The effect of systemic isotretinoin therapy on procedural wound healing remains debated due to the lack of high‐quality evidence. There is one report in the literature regarding delayed wound healing in extrafacial cannular entry sites in a patient on oral isotretinoin [22]. Concomitant use of isotretinoin and subcision has not been studied in the literature; however, the most recent studies have suggested that dermatological surgery in patients on isotretinoin has shown no increased side effects [23]. The absence of evidence of harm cannot be equated with evidence of no harm, and hence the dermatologic surgeon should consider factors such as isotretinoin dose, family/personal history of scarring, as well as operative factors such as aggression of undermining, and other energy‐based procedures that maybe performed concurrently.

## 6 Conclusion

Subcision is a commonly used treatment method for revision of acne scarring. Since the invention of subcision, many modifications—mainly in the form of new subcision tools—have been made to improve the efficacy and precision. In terms of complications, subcision is associated with often transient bruising, hematoma, dyspigmentation, and subcutaneous lumps at the subcision site. Hematoma and infection are rare complications of subcision. Although hypertrophic scarring was reported to be a complication in the original study, it is seldomly seen within the literature. The current literature is dearth in terms of direct comparative studies between different subcision tools and their safety profile. More higher‐powered comparative studies are needed to further elucidate the difference in complication rate between various subcision equipment that are available in clinical practice.

## Author Contributions

Study conception and design by Davin Lim. Drafting of the manuscript and analysis by Cong Sun.

## Ethics Statement

The study protocol conformed to the ethical guidelines of the 1975 Declaration of Helsinki.

## Conflicts of Interest

The authors declare no conflicts of interest.

## Data Availability

Data sharing not applicable to this article as no datasets were generated or analysed during the current study.
